# Pharmacokinetics and Pharmacodynamics Evaluation of Tramadol in Thermoreversible Gels

**DOI:** 10.1155/2017/5954629

**Published:** 2017-07-27

**Authors:** Juliana Zampoli Boava Papini, Cíntia Maria Saia Cereda, José Pedrazzoli Júnior, Silvana Aparecida Calafatti, Daniele Ribeiro de Araújo, Giovana Radomille Tofoli

**Affiliations:** ^1^São Francisco University, Av. São Francisco de Assis 218, 12916-900 Bragança Paulista, SP, Brazil; ^2^Institute and Research Center São Leopoldo Mandic, Rua José Rocha Junqueira 13, 13045-75 Campinas, SP, Brazil; ^3^Federal University of ABC, Rua Santa Adélia 166, 09210-170 Santo André, SP, Brazil

## Abstract

We evaluated pharmacokinetics (PK) and pharmacodynamics (PD) induced by new formulations of tramadol (TR) in thermoreversible gels. The poloxamer- (PL-) tramadol systems were prepared by direct dispersion of the drug in solutions with PL 407 and PL 188. The evaluated formulations were as follows: F1: TR 2% in aqueous solution and F2: PL 407 (20%) + PL 188 (10%) + TR 2%; F3: PL 407 (25%) + PL 188 (5%) + TR 2%; F4: PL 407 (20%) + TR 2%. New Zealand White rabbits were divided into four groups (*n* = 6) and treated by subcutaneous route with F1, F2, F3, or F4 (10 *μ*g·kg^−1^). PK evaluation used TR and M1 plasma levels. PD evaluation was performed with the measurement of both pupils' diameters. F2 showed higher TR plasma concentration after 180 minutes and presented lower M1 concentrations at almost all evaluated periods. Areas under the curve (ASC_0–480_ and ASC_0–*∞*_) and clearance of F2 presented differences compared to F1. F2 presented significant correlation (Pearson correlation) between the enhancement of TR and M1 concentrations and the decrease of pupil size (miosis). Thus, F2 was effective in altering pharmacokinetics and pharmacodynamics effects of TR.

## 1. Introduction

Tramadol (TR) is an opioid analgesic widely used to treat moderate, severe, and chronic pain, such as oncologic and postoperatory pain [1, 2]. It acts as an opioid *μ*1 receptor agonist and monoamine reuptake inhibitor and as a target for some protein coupled receptor and ligand-gated ion channels [3, 4]. The common adverse effects of tramadol are somnolence, seizures, nausea, and vomiting [4]. TR usually evokes a low incidence of adverse effects when compared to classical opioids, such as morphine and fentanyl [5]. Despite these advantages, TR presents short duration of action and it is necessary to make repeated doses or continuous infusion for a prolonged analgesic action [6, 7].

In this context, our research group developed drug deliveries systems with TR and poloxamer (PL) thermoreversible hydrogels for future treatment of postoperatory pain [5]. Poloxamers are copolymers composed of basic units of ethylene oxides and propylene oxides. The different number of these basic units in PL allows the formation of micelles with a hydrophobic core surrounded by a hydrophilic corona. PL have the ability, in concentrated solutions, of forming gels close to corporal temperatures because when the temperature rises, propylene oxides units are dehydrated and aggregate (micellar core), while the hydrophilic ethylene oxides units (micellar corona) remain hydrated. Thus, in low temperatures the system remains as fluids and this property can be used for parenteral administration of drugs and in high temperatures (close to corporal) it remains as semisolids and allows drug delivery for long periods of time [8–10].

Physicochemical aspects, dissolution-release profiles, cytotoxicity, genotoxicity, and in vivo pharmacological performance of poloxamer- (PL-) based binary hydrogels were studied by our research group and results were showed at the work of dos Santos and colleagues (2015) [5]. In this study, TR (20 mg·mL^−1^) was dispersed in different solutions containing PL 407 alone or in binary systems with PL 188. Physicochemical characterization showed that the formation of binary systems composed of PL 407 and PL 188 alters the micellization and sol-gel transition processes. The temperature of micellization temperature (*T*_*m*_) for the binary system was nearby 11 to 12°C and presents discrete variation when compared to PL 407 hydrogels (*T*_*m*_ about 9–14°C). The sol-gel transition temperature (*T*_sol-gel_) was lower for PL 407 (from 22 to 24°C) in high concentrations (30 and 35%, w/w%) which enabled the use of this systems for parenteral injection. The PL 407 system (20%) presents *T*_sol-gel_ around 30°C. The PL 407–PL 188 binary systems (20 : 10 and 25 : 5) showed Tsol-gel in a range of 32°C–38°C; thus in temperatures close to corporal these systems remain as semisolids. For TR solution 100% of release was achieved after 4 hours. The release profiles of TR in the formulations with PL 407 (20%) and the PL 407–PL 188 binary systems (20 : 10 and 25 : 5) over 24 hours were 65.6% ± 1.4%; 72.6% ± 8.6%; and 45.1% ± 2.5%, respectively. The formulations with PL reduced the cytotoxicity compared to TR and did not present genotoxic effects. Analgesic activity assay demonstrated that PL 407 and its binary systems with PL 188 are effective hydrogels for controlling and prolonging TR release for 48–72 hours after subcutaneous injection.

Data obtained by dos Santos et al. (2015) [5] supported the advantages of the association of TR in poloxamers hydrogels. Among the various formulations tested by dos Santos and coworkers (2015) [5] we select three of them which presented the best performance regarding physicochemical aspects, cytotoxicity, and in vivo pharmacological effect for an in vivo evaluation. Thus, the purpose of this study was to evaluate the preclinical pharmacokinetics (PK) and pharmacodynamics (PD) induced by these new formulations of TR in thermoreversible gels to support its future clinical use.

## 2. Material and Methods

### 2.1. Chemicals and Reagents

TR hydrochloride (attested purity of 98.5%) was donated by Cristália Produtos Químicos Farmacêuticos Ltda. (Itapira, Brazil). O-Desmethyltramadol (M1), poloxamer 407 (Pluronic® F127), and poloxamer 188 (Pluronic F68) were purchased from Sigma-Aldrich Co. (St Louis, MO, USA). All other reagents were of analytical grade and deionized water from a PURELAB Option-Q (ELGA LabWater, High Wycombe, UK) water system was used for all experiments.

### 2.2. Hydrogels Preparations

The formulations used in this study were as follows: F1: TR 2% in aqueous solution; F2: PL 407 (20%) + PL 188 (10%) + TR 2%; F3: PL 407 (25%) + PL 188 (5%) + TR 2%; and F4: PL 407 (20%) + TR 2%. For F2, F3, and F4 the hydrogels were prepared in the same conditions as described by dos Santos et al. (2015) [5]. TR (2%) was dispersed in different solutions containing PL 407 alone or with PL 188 at 4°C under magnetic stirring (100 rpm). The PL concentrations were selected in order to obtain the three formulations tested in our study.

### 2.3. Animal Protocol: PK-PD Study

The experimental protocol was approved by the Institutional Committee for Ethics in Animal Research of São Francisco University (protocol number 002.04.2013). Animals were housed 1 per cage and received water and food ad libitum with a 12:12 hours' light-dark cycle, at 23 ± 2°C. This randomized blind study was conducted with 24 New Zealand White rabbits (2.50–3.00 kg) divided into four groups (*n* = 6). Animals were treated by subcutaneous route with one of the formulations described above (10 *μ*g·kg^−1^). The TR dosage was based on previous work of Souza and coworkers (2008) [11] and the recommendations of Barter (2011) [12], and also the dosage was evaluated in a pilot study (data not shown). Rabbits received the injection in the subcutaneous tissue in the unattached skin around their neck and the needle was a 25 G × 1 in. (BD®). The needle was inserted with a 45° angle.

An intravascular catheter was inserted in the ear vein of the animals and blood samples (2 mL) were collected via a heparinized cannula before dose (0 min) and at 15, 30, 45, 60, 90, 120, 180, 240, 300, 360, 420, and 480 minutes after the injection of formulations. These intervals were defined to provide ten samples between the base line (0 min) and approximately 4 times *t*_1/2_ (half-life time) of TR (approximately 2 h) [11]. Immediately after each blood collection plasma was separated and stored at −70°C until analysis [13].

In order to assess the efficacy of these new formulations, animals had their pupils size assessed in millimeters based on a digital calliper (Digimatic Calliper, Mitutoyo, Tokyo, Japan) at the same periods of blood sample collection. Both pupils were measured and the mean value was used as reference. The measurements occurred in the same location and under similar brightness of light at all evaluation times [14].

### 2.4. LC-MS/MS Assay: Apparatus and Chromatographic Conditions

A Shimadzu LC 20 AD system coupled with a Micromass Quattro LC® triple stage quadrupole mass spectrometer (LC-MS-MS), equipped with an API (Atmospheric Pressure Ionization) electrospray source, was used to determine the TR and O-desmethyltramadol (M1) plasma levels.

The chromatographic conditions were determined after validation of the analytical method for TR and M1. In order to validate the method, quality control samples of TR (QC: 2400.0, 1200.0, and 6.0 ng·mL^−1^) were prepared by mixing drug-free plasma with appropriate volumes of working solutions. For M1 we used QC samples in different concentration as follows: 40.0, 25.0, and 3.0 ng·mL^−1^.

TR analytical method used a Synergi Fusion (150 × 2 mm id, 4 *μ*m particle size) for all separation instances. The mobile phase was 85% acetonitrile and 15% water with 0.1 mL of formic acid (pH = 3.5). The total run time was 3.5 minutes; retention time for TR was 0.72 min. The mass spectrometer was run in the positive mode (ES+) and set for multiple reaction monitoring (MRM). The full-scan single-mass spectrum and the daughter ion-mass spectrum for TR and diazepam (internal standard, IS) were (*m*/*z*) 264.14 > 58.28 and 285.20 > 193.00, respectively. Sample preparation for TR was carried out after frozen plasma samples (200.0 *μ*L) were thawed at room temperature, followed by the addition of 50 *μ*L of IS work solution (5 *μ*g·mL^−1^). One thousand microliters of dichloromethane (1 : 1; V/V) was added and then the sample was vortexed for five minutes and centrifuged at 1200 ×g, for 10 min at −4°C. The organic liquid (0.7 *μ*L) layers were transferred to microtubes and the samples were dried under nitrogen flow, samples were reconstituted in 200 *μ*L mobile phase, vortexed for three minutes, and 150 *μ*L was transferred to LC-MS/MS system vials, for further injection (5.0 *μ*L).

For M1 detection all separation instances were carried out with a C18 Luna (100 × 6 mm id, 5 *μ*m particle size). The same volume of frozen plasma samples (200 *μ*L) were thawed at room temperature and also 50 *μ*L of internal standard (IS) (diazepam, 200 ng·mL^−1^) was added. The other procedures were the same used in TR quantification. But, for M1, the samples were reconstituted in 100 *μ*L mobile phase (acetonitrile and ammonium acetate (5 mM); 95 : 5 V/V). The total run time was 5.0 minutes; retention time for M1 was 1.48 min. The full-scan single-mass spectrum and the daughter ion-mass spectrum for M1 were (*m*/*z*) 250.64 > 58.50.

The data were integrated using the MassLynx 4.1 (Waters®) software in both analytical methodologies. Precision and accuracy of the analytical method were controlled by calculating the intrabatch and interbatch variation at three concentrations of QC in five replicates (*n* = 5). Three calibration curves were plotted in the range of 2.0–3000.0 ng·mL^−1^ for TR and for M1 the range was 2.0–3000.0 ng·mL^−1^. The limit of quantification (LQ) was defined as the lowest concentration at which precision and accuracy were within 20% of the true value for both M1 and TR.

### 2.5. Statistical Analysis

The concentration-time data were analyzed by the noncompartmental approach. The pharmacokinetic parameters were calculated using WinNonlin software (WinNonlin version 5.3, Pharsight Corporation, CA, USA). The plasma TR and M1 concentrations were analyzed by one-way ANOVA and the Tukey-Kramer test (post hoc) considering each period of time separately (*α* = 0.05). The pharmacokinetic parameters of both compounds were also analyzed using one-way ANOVA and the Tukey-Kramer test (post hoc) (*α* = 0.05). Plasma concentrations of TR and M1 were correlated with the pupil's diameters using Pearson correlation. For the analysis we used GraphPad InStat and Prism (GraphPad Software, Inc., La Jolla, CA, USA).

### 2.6. Results and Discussion

In the present study we aimed to evaluate preclinical pharmacokinetics and pharmacodynamics of a new TR formulation. Preclinical evaluation is an important (and mandatory) part on new formulations development, since the in vitro results may not be reproducible during in vivo studies. Rabbits are good options to perform pharmacokinetic studies, especially because these animals present a higher volume of blood and easy ways to collect it when compared to rats. Their ear vein can be easily cannulated with a simple puncture technique to collect multiple plasma samples. Also, we decided to use “large experimental animal models” to observe extensive whole-body pharmacokinetics in a context comparable to patient physiology [15, 16]. In our study, we also evaluated M1, the main TR metabolite, levels and its PK parameters since it has about 300-fold higher affinity for the *μ* receptor than TR [4].

In order to evaluate the pharmacokinetics parameters we have to determine TR and M1 concentration in plasma. To achieve this goal we developed an analytical methodology which presented reliable and reproducible results within its analytical range for both TR and M1. The analysis of TR and M1 did not present neither interfering compounds nor ion suppression. The assays were linear for TR and M1 and coefficients of correlation (*r*) were greater than 0.99 for all the calibration curves (TR *r* values: 0.997; 0.996; 0.998; M1 *r* values 0.996; 0.994; 0.995). Intra- and interbatch accuracy of QC TR plasma samples ranged from 85.14 to 109.03% and precision ranged from 1.05 to 5.58%. M1 accuracy and precision ranged from 98.18 to 111.86% and from 4.32 to 17.44%, respectively. The LQ for TR was 2.00 ng·mL^−1^ and for M1 was 1.00 ng·mL^−1^.

**Figure 1:**
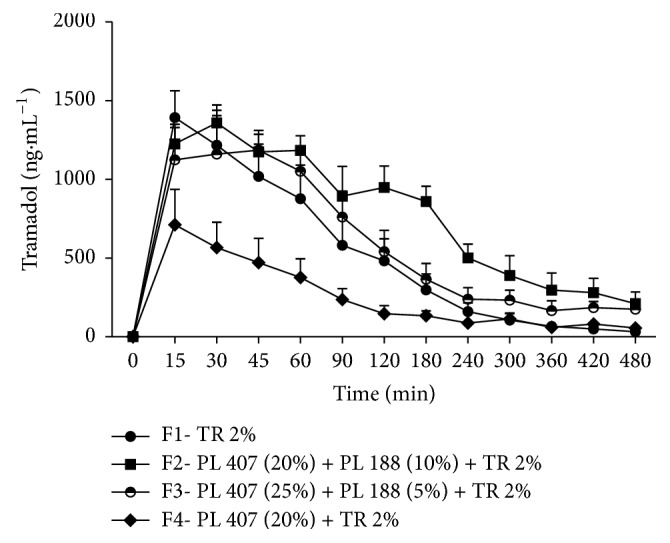
Time-course (min) after the injection of TR formulations in rabbits. Values are expressed as mean ± SD. F4 < F1 and F2 and F3 after 30 to 60 min (*p* < 0.05); F4 < F2 and F3 after 90 min (*p* < 0.05). F2 > F1 and F4 after 120 min and after 180 min F2 > F1 and F3 and F4 (*p* < 0.05).

After 15 minutes of the subcutaneous administration of all the formulations in rabbits, all animals presented TR in the systemic circulation. Thirty to sixty minutes after the injections F4 (PL407 (20%) + TR 2%) presented lower plasma concentrations when compared to all other formulations (F1: TR 2%; F2: PL 407 (20%) + PL 188 (10%) + TR 2%; and F3: PL 407 (25%) + PL 188 (5%) + TR 2%) (*p* < 0.05). F4 still presented lower concentrations than F2 and F3 (*p* < 0.05) after 90 minutes. After 120 minutes, F2 presented higher concentrations than F4 and F1 and 60 minutes later the differences were among F2 and all the other formulations (*p* < 0.05). Two hundred and forty minutes after the injections there were no differences between all tested formulations (Figure 1).

**Figure 2:**
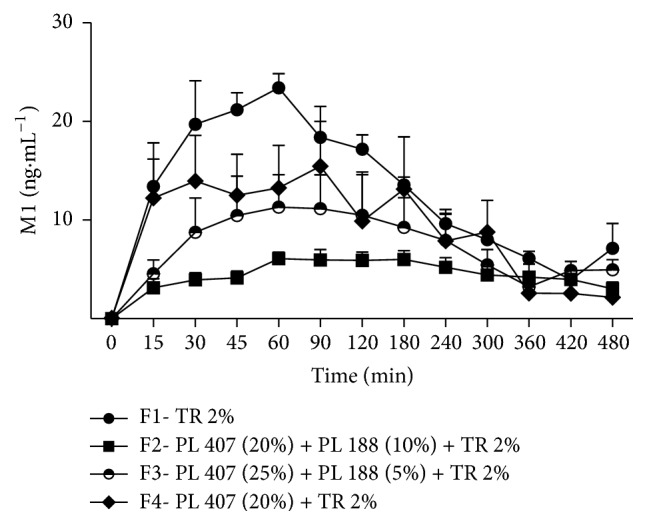
Time-course (min) of M1 after the injection of TR formulations in rabbits. Values are expressed as mean ± SD. F2 < F1 until 120 minutes (*p* < 0.05); F4 < F2 after 15, 30, and 90 minutes (*p* < 0.05).

M1 plasma concentrations were lower when TR concentrations were higher and vice versa. For example, F2 presented lower M1 concentrations and higher TR concentrations at almost all evaluated periods. M1 concentrations after the injection of F2 were lower than F1's until 120 minutes after the injection. F4 presented higher concentrations for M1 when compared to F2 at 15, 30, and 90 minutes after the injections (*p* < 0.05) (Figure 2).

Maximum plasma concentration (*C*max) of F4 was approximately 50% smaller and volume of distribution (*V*_*d*_) was two to three times higher than the others formulations (*p* < 0.05). F2 values for areas under the curve (ASC_0–480_ and ASC_0–*∞*_) presented differences between F1 and F4 (*p* < 0.05). Clearance (CL) of F2 was approximately half of F1 (*p* < 0.05). *T*max (time to reach maximum concentration) and *t*1/2 (half-life time) did not show any statistical differences between the formulations. MRT (Mean residence time) values for F4 were twice the values for F1 (*p* < 0.05) (Table 1). *t*1/2, *T*max, and MRT of M1 did not present any statistical differences. *C*max and AUC_0–*∞*_ values of M1 for F1 were higher than F2 (*p* < 0.05). *V*_*d*_ was higher for F2 than F1 (*p* < 0.05) (Table 2).

Formulations F2 and F4 still presented TR in plasma after 480 minutes. In our study it was not possible to prolong the period of blood removal, because of the total blood volume that can be removed without interfering with the normal homeostasis and consequently with the PK parameters. The same removed blood volume was replaced with warm saline solution. Rabbits present a volume of circulating blood around 44–70 mL·kg^−1^ and removal of approximately 10% of the circulating blood volume will initiate homeostatic cholinergic mechanisms, if 15–20% volume is removed cardiac output and blood pressure will be reduced, and more than 40% loss can cause haemorrhagic shock [17]. During the pilot study (data not shown) we established the restraint method for animals. The rabbits were restrained in a plastic restrainer designed for rabbits (Insight Ltda., Ribeirão Preto, SP, Brazil) for two hours. During this period the TR formulations kept the animals slightly sedated. After 120 minutes the animals were placed in cages and had free access to water and food. Animals were restrained only during blood removal. Despite the fact that we do not collect blood after 480 minutes, we still observe alterations in PK parameters of TR formulations.

Extended release formulations can produce distinct PK profiles and drug-release pattern to provide a drug concentration in a sustained or controlled manner. These formulations might produce a lag time in drug absorption or present a plasma concentration with a sharp initial slope followed by a sustained release phase. In both cases, the fluctuations in plasma concentrations of the drug associated with unpredictable effects of the conventional formulations can be avoided [18].

In our study, F2 (PL 407 (20%) + PL 188 (10%) + TR 2%) and F4 (PL 407 (20%) + TR 2%) presented features that can be observed in typical drug-delivery formulations. F4 presented more constant and lower TR plasma concentrations in almost all periods of time and a small *C*max and a higher MRT when compared to the free drug. These alterations (reduced absorption and maintaining constant drug concentration) are similar to the in vitro findings for this formulation (release of 65% of TR in 24 hours) [5]. Also these findings are similar to the pattern observed by commercial formulation of sustained release of TR, Zytram XL®, available at USA and Canada for oral administration. The pharmacokinetic profile of a 200 mg tablet Zytram XL shows that the *C*max was 34% lower when compared to a 100 mg dose of Tramadol given as an oral solution [18]. However, our formulation, F4, did not present differences in *T*max and *t*1/2.

F2 produced higher and more constant concentrations than the other formulations and after 180 minutes it still presents higher plasma levels. Also F2 was effective in enhancing the bioavailability (higher AUCs values) and in reducing the TR clearance. These alterations are similar to the pattern observed by commercial formulation Tridural® available at Canada for oral administration. In both situations it is possible to observe a sharp initial slope followed by a sustained release phase and a higher bioavailability [18]. The previous work from our group showed a rapid dissolution profile for the formulations used in our study, which is important to allow drug release to the site of action. Also, the in vitro release evaluation for these formulations showed slow release of TR (around 65 to 70% in 24 hours). The dissolution/release relationship showed was effective in controlling and prolonging TR release in vitro [5]. In our study this same feature produced the sharp initial slope followed by a sustained release phase and a higher bioavailability observed for F2.

Usually, efficacy of opioids is demonstrated based on their antinociception and analgesic activity [19]. However, in large animals models, like the one used in our study, this can be difficult to achieve. The degree of pain in rabbits can vary importantly between animals and there are no objective criteria for this evaluation. As a prey species, rabbits may hide their pain by remaining motionless. Thus, rabbits appear to respond to pain in an opposite fashion of mice or rats and have little activity or behaviour to be assessed [12]. The lack of pain behaviour in rabbits leads to the use of pupil size to determine the opioid efficacy. Pupil size can be used to determine the biologic effects of opioids [20–22], since these drugs produce miosis in rabbits [23].

**Figure 3:**
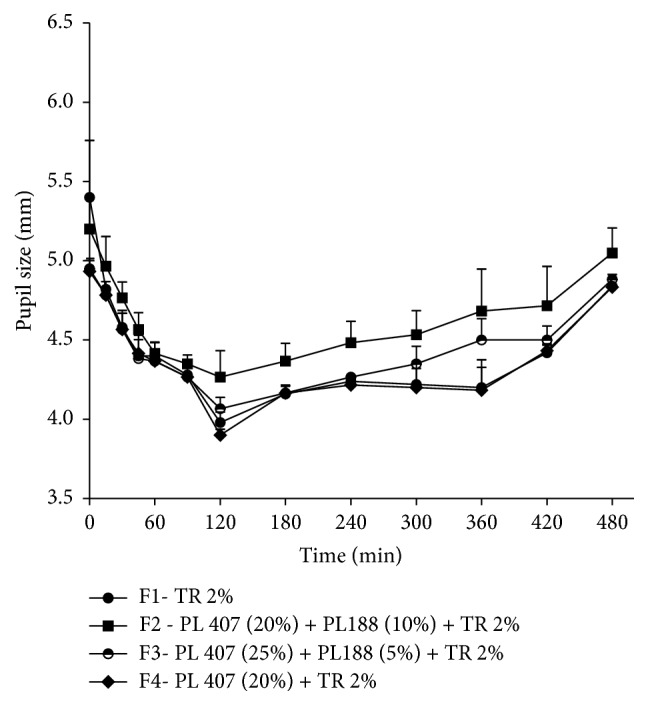
Time-course (min) of pupil size after the injection of TR formulations in rabbits. Values are expressed as mean ± SEM.

**Figure 4:**
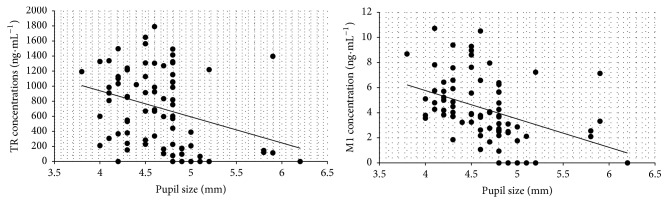
Correlation of TR and M1 plasma levels with miosis after subcutaneous administration of F2.

Pupil size values were statistically analyzed separately in a three-way fashion. First, initial values of all animals were compared to observe regularity between the animals. The basal values (5.08 ± 0.21 mm) did not present statistical differences among the four tested groups (*p* > 0.05; ANOVA/Tukey-Kramer). Second, a time-course of pupil size variations was analyzed (Figure 3). After 30 minutes F3 promoted smaller pupil size when compared to the basal measures before the injections (*p* > 0.05; ANOVA/Tukey-Kramer). Considering F2 and F4 this difference occurred only after 45 minutes (*p* > 0.05; ANOVA/Tukey-Kramer). Our previous work reported that time for hydrogel formation was less than 20 s, and this can explain the longer onset time for the depot formulations. However, this onset time is not a disadvantage and is still comparable with oral TR formulation that shows the disadvantage of short duration of action (from 3 to 6 hours) [24]. Finally, we correlated (Pearson correlation) each measurement of pupil's size with TR and M1 plasma concentration in order to evaluate if the enhancement of plasma concentrations of TR and M1 evoked biologic effect. Correlation was observed only with F2, which promoted TR plasma concentrations weak correlation with pupil's size (*r* = −0.315), and M1 concentration presented moderate correlation (*r* = −0,409). These results indicate that the boost of TR and M1 plasma concentration is correlated with the occurrence of miosis in rabbits after F2 administration (Figure 4).

In our study it is not possible to claim that the formulations with PL prolonged the duration of TR effect based on pupil size, since all animals exhibited reduction in pupil size until 480 minutes and we were not able to prolong that assessment as reported above. But the analgesic duration profile was evaluated in a well-established and accepted model in rats in our group previous work [5] and our goal was to correlate plasma concentration of TR and M1 with biologic effect. This is important to show that the slow release is not in such a low velocity/intensity that no biologic effect would be observed. Our results suggested that the in vivo slow release of TR can produce sufficient plasma levels to evoke biologic effect.

### 2.7. Conclusion

Drugs prescribed for the management of chronic pain should present fast onset and regular absorption, as well as significant plasma levels to be able to provide adequate pain relief. TR presents short duration of action [5] and our group intended to develop a new formulation to overcome this feature. In our study we were able to prove that these new formulations in fact modified the release of TR in vivo. One formulation (F2: PL 407 (20%) + PL 188 (10%) + TR 2%) presented fast onset (observed with pupil size) and high plasma levels at the end of the dosing interval. Thus, the association of PL 407 (20%) and PL188 (10%) in formulation 2 (F2) was effective in altering pharmacokinetics and pharmacodynamics effects of TR. F2 was effective in enhancing the bioavailability and this effect was correlated with a more intense biologic effect. These results, associated with the lack of cytotoxic effects of the used PL combinations [5], encourage the use of this new formulation as a safe and effective option for subcutaneous application of TR.

## Figures and Tables

**Table 1:** Tramadol pharmacokinetics parameters: *t*1/2, *C*max, AUC_0–480_, AUC_0–*∞*_, *T*max, CL, *V*_*d*_, and MRT after the injection (SC) of F1, F2, F3, and F4 in rabbits. Data expressed as mean (±SD).

	F1	F2	F3	F4
*t*1/2 (h)	1.41 ± 0.31	2.46 ± 0.86	2.35 ± 1.28	7.55 ± 11.52
*T*max (h)	0.45 ± 0.20	0.750 ± 0.671	0.54 ± 0.18	1.83 ± 2.45
*C*max (ng·mL^−1^)	1563.82 ± 404.77	1452.95 ± 200.34	1374.93 ± 314.63	756.81 ± 490.89^b*∗∗*,c*∗*,d*∗*^
AUC_0–480_ (ng-h·L^−1^)	2581.86 ± 1417.41	4971.81 ± 1695.77^a*∗*,c*∗∗∗*^	3320.01 ± 1445.91	1307.32 ± 704.39
AUC_0–*∞*_ (ng-h·L^−1^)	2658.33 ± 1498.56	5894.02 ± 2791.82^a*∗*,c*∗∗*^	4533.81 ± 1267.25	1992.07 ± 117.42
*V*_*d*_ (L)	10.13 ± 6.01	6.24 ± 0.70	7.14 ± 2.31	16.81 ± 2.48^b,*∗*c*∗∗∗*,d*∗∗*^
CL (L·h^−1^)	5.33 ± 3.62	1.95 ± 0.69^a*∗*^	2.41 ± 0.97	5.03 ± 0.29
MRT (h)	1.65 ± 0.60	2.72 ± 0.42	2.66 ± 0.71	2.96 ± 1.18^b*∗*^

Statistical analysis: ^a^F1 versus F2; ^b^F1 versus F4; ^c^F2 versus F4; ^d^F3 versus F4; *p* < 0.001 [*∗∗∗*], *p* < 0.01 [*∗∗*], and *p* < 0.05 [*∗*], ANOVA/Tukey-Kramer.

**Table 2:** Pharmacokinetics parameters *t*1/2, *C*max, AUC_0–480_,  AUC_0–*∞*_, *T*max, CL, *V*_*d*_, and MRT of M1 after the injection (SC) of F1, F2, F3, and F4 in rabbits. Data expressed as mean (±SD).

	F1	F2	F3	F4
*t*1/2 (h)	2.64 ± 0.86	4.55 ± 1.44	4.02 ± 2.48	5.57 ± 8.36
*T*max (h)	0.70 ± 0.27	2.70 ± 2.04	2.95 ± 2.70	1.58 ± 1.75
*C*max (ng·mL^−1^)	27.00 ± 2.69^a*∗∗*^	7.82 ± 2.23	15.17 ± 9.96	21.89 ± 11.81
AUC_0–480_ (ng-h·L^−1^)	89.28 ± 5.26	37.82 ± 11.93	56.18 ± 39.59	65.79 ± 50.29
AUC_0–*∞*_ (ng-h·L^−1^)	121.71 ± 34.00^a*∗*^	48.92 ± 6.31	109.06 ± 53.35	83.57 ± 53.82
*V*_*d*_ (L)	310.97 ± 53.25^a*∗∗∗*^	1325.73 ± 320.55	611.87 ± 379.42	286.80 ± 96.56
CL (L/h)	86.71 ± 20.70	207.10 ± 26.48	120.02 ± 81.57	111.78 ± 48.61
MRT (h)	2.99 ± 0.26	3.71 ± 0.43	3.85 ± 1.23	3.02 ± 1.02

Statistical analysis: ^a^F1 versus F2; *p* < 0.001 [*∗∗∗*], *p* < 0.01 [*∗∗*], and *p* < 0.05 [*∗*], ANOVA/Tukey-Kramer.
